# Take-home naloxone (THN) ownership among a community-based cohort of sex workers who use drugs in metro vancouver: longitudinal trends and structural determinants (2017–2024)

**DOI:** 10.1186/s12954-026-01483-1

**Published:** 2026-06-11

**Authors:** Sarah Moreheart, Kate Shannon, Andrea Krüsi, Wiebke Bartels, Haoxuan Zhou, Kanna Hayashi, Emily Luba, Colette Merrison, Shira Miriam Goldenberg

**Affiliations:** 1 Faculty of Health Sciences, Simon Fraser University, 8888 University Drive, Burnaby, BC V5A 1S6 Canada; 2 Department of Medicine, University of British Columbia, 2755 Laurel Street, Vancouver, BC V5Z 1M9 Canada; 3 School of Population and Public Health, University of British Columbia, 2206 East Mall St, Vancouver, BC V6T 1Z3 Canada; 4 Department of Criminology, Faculty of Arts, Simon Fraser University, 8888 University Drive East, Burnaby, BC V5A 1S6 Canada; 5 BC Centre on Substance Use, 400-1045 Howe Street, Vancouver, BC V6Z 2A9 Canada; 6 Division of Epidemiology and Biostatistics, School of Public Health, San Diego State University, 5500 Campanile Drive, San Diego, CA 92182- 4162 USA; 7 Division of Infectious Diseases and Global Public Health, University of California San Diego, 9500 Gilman Drive, La Jolla, CA 92093-0507 USA

**Keywords:** Sex workers, Drug toxicity crisis, Take-home naloxone (THN), Overdose prevention

## Abstract

**Background:**

Sex workers who use drugs face disproportionately high overdose burden, driven by drug toxicity and intersecting structural factors in sex work and drug use environments. Given limited evidence regarding sex workers’ use of overdose prevention tools, including take-home naloxone (THN), we examined period prevalence, time trends, and structural factors longitudinally associated with THN ownership among sex workers over 7 years.

**Methods:**

Data derived from An Evaluation of Sex Workers’ Health Access, a prospective, community-based cohort of sex workers in Vancouver, Canada (March 2017–March 2024). Generalized linear mixed-effects modeling (GLMM) examined structural factors associated with THN ownership.

**Results:**

Among 506 participants, 90.3% (*N* = 457) ever owned THN. Period prevalence of THN ownership remained high across semi-annual visits( 72.6–88.8%). In multivariable GLMM modeling, THN ownership was associated with injection drug use (Adjusted Odds Ratio [AOR] 1.70, 95% Confidence Interval [CI] 1.35–2.14), enrollment in opioid agonist therapy (AOR 1.60, 95% CI 1.29–1.98), and drug use with sex work clients (AOR 1.27, 95% CI 1.02–1.59); whereas recent homelessness was associated with lower odds of THN ownership (AOR 0.52, 95% CI 0.37–0.73). Having used sex work-specific programs was marginally associated with higher odds of THN ownership.

**Conclusion:**

The majority of sex workers in this study owned THN, with high prevalence sustained over time. Ownership was particularly associated with enrollment in opioid agonist therapy and drug use with clients. However, recent homelessness was a barrier to THN ownership. Findings highlight the importance of integrating THN distribution and training into sex work-specific programs, with a focus on enhancing access to treatment and addressing structural factors that impact both safety and overdose prevention. Ensuring sex workers’ right to health via strengthening THN access and equity-driven harm reduction strategies that prioritize the most marginalized sex workers is recommended.

## Introduction

The enduring overdose crisis driven by the toxicity of the unregulated drug supply has highlighted the devastating impact of prohibitionist drug policy among people who use illicit drugs, as evidenced by the over 12,000 deaths in British Columbia (BC) since declaration of the public health emergency in April 2016 (BC Coroners Service, [1]). To prevent overdose death in community settings, timely community-based administration of take-home naloxone (THN) is critical [2, 3]. However, to be effective, community members must have access and training to THN, which are typically distributed by community-based health and harm reduction services [4]. Current research conducted with people who use drugs suggests access to overdose prevention education and naloxone distribution services may be inequitable, especially among those who experience structural inequities such as gendered violence or homelessness (5–7). Sex workers who use drugs experience disproportionate health and social inequities and heightened structural barriers to services due to the stigmatization and marginalization of both sex work and drug use [5–8]. Responses to address these inequities require tailored, gender-specific interventions [6, 9]; Harris et al., [10]. Despite the high prevalence of sex workers who use drugs globally [11], little has been documented on the overdose prevention needs and access of this population.

THN is a tool to address overdose-related morbidity and mortality in the context of a witnessed opioid overdose event [12] and is a well-established part of evidence-based secondary/tertiary prevention interventions which aim to minimize these acute consequences [13, 14]. THN access and utilization have been identified as critical steps for facilitating effective overdose prevention among people who use drugs, including the need for sufficient awareness, obtainability, availability, training, and ownership/possession [15]. In comparison to a dominant emphasis on substance use treatment interventions [14, 16, 17], access and scale-up of life-saving overdose response tools such as THN is needed to address the immediate risk of overdose-related morbidity and mortality amid the current overdose crisis [3, 18–21]. THN ownership represents more beyond a harm reduction behavioural outcome. It serves as a structural marker on inclusion in harm reduction care and intervention programmes within a criminalized drug environment and the realization of the right to survive preventable overdose [12]. In BC, THN kits contain ampoules of naloxone with a needle to inject, and are available at no-cost [22]. Facilities approved for distribution are provided with resources to facilitate THN administration training to accompany the hand-out of THN kits to individuals [22]. While assessment of the reach of THN is needed to ensure these programs are reaching impacted populations [15, 23, 24], to our knowledge this has not yet been done for sex workers who use drugs.

Barriers to health and harm reduction service access have been extensively documented for sex workers and highlight the role of structural factors within both sex work and drug use risk environments that can either positively or adversely shape the health and safety of sex workers who use drugs. Many studies focus on HIV and overdose-related risks, including gender-based violence [25] and drug-related gendered power dynamics [26–29] across both occupational and intimate partner contexts. Additional factors include the disproportionate policing and criminalization of both sex work and drug use [7, 30]; living conditions (e.g., precarious housing) [31]; structural stigma [32], and engagement with community and peer-based supports [8, 33–36]. Criminalization of sex work, criminalization of drug use, gender-based violence, alongside precarious housing access, and racialized policing do not operate independently, but rather are interlocking systems of structural power that work together to produce heightened overdose vulnerability and uneven access to life-saving harm reduction interventions among sex workers who use drugs [6]. These intersecting inequities reflect systemic violations of sex workers’ human rights, including rights to health, safety, and non-discriminatory access to supports and services.

While structural factors are increasingly recognized as critical for shaping marginalized populations’ health, much previous work on the overdose crisis has focused on individual and behavioral factors, including unmet mental health needs [37], and substance use treatment outcomes [38]. There is limited existing research on how structural factors within both sex work and drug use environments influence the utilization of supports aimed at mitigating drug-related harms including overdoses, such as THN, among sex workers who use drugs [7, 39–41].

Recognizing the important role that structural determinants within the sex work and drug use environment play in access to health and harm reduction services, our study was guided by a conceptual framework informed by the structural determinants of sex worker health [8, 42] and the risk environment for drug-related harm [43, 44]. Our framework considers both risk and protective factors and focuses attention on the role of structural determinants on THN ownership among sex workers. Given the stark health inequities - including the high rates of non-fatal overdoses - among sex workers who use drugs [45, 46], examining access to THN is critical to understanding how life-saving interventions are differentially realized within structurally marginalized populations. Such evidence is needed to inform structural interventions that enable accessible, sex-worker friendly overdose prevention strategies. Despite extensive evidence supporting THN effectiveness, little longitudinal research has examined how access evolves over time within criminalized and structurally marginalized populations. Longitudinal analyses are essential to capturing how shifting policy environments, overdose crisis intensification, housing instability, and service expansion efforts shape patterns of THN ownership. This study aimed to examine (1) period prevalence and time trends in THN ownership, and (2) the association between structural determinants in the sex work and drug use environment and THN ownership among sex workers who use drugs over 7 years (2017–2024).

## Methods

Data were derived from An Evaluation of Sex Workers Health Access (AESHA), a prospective, open cohort of indoor, street-based, and online-advertising sex workers which operated from 2010 to 2024. This study was developed based on collaborations with local sex work agencies since 2005 [47] and continues to be monitored by a Community Advisory Board (CAB) of representatives of 15 + community agencies. Eligibility criteria at enrolment included identifying as a cisgender or transgender woman^1^ over 14 years of age, exchanging sex for money within the last 30 days, and able to provide written informed consent. Time-location sampling was used to recruit youth and adult cisgender and transgender women engaged in recent sex work through day and late-night outreach to outdoor/public sex work locations (i.e., streets, alleys) and indoor sex work venues (i.e., massage parlours, micro-brothels, and in-call locations) across Metro Vancouver [47]. In addition, online recruitment was used to reach sex workers in online solicitation spaces. Indoor sex work venues and outdoor solicitation spaces (‘strolls’) were identified through community mapping conducted together with current/former sex workers and continued to be updated by the outreach team. The study holds ethical approval through the Providence Health Care/University of British Columbia Research Ethics Board.

### Data collection

As questions on take-home naloxone access were added in 2017, analyses were restricted to participants who used drugs and completed semi-annual questionnaire data collected between March 2017-March 2024. Questionnaires were administered by trained interviewers/outreach workers with lived (i.e., current/former sex work) and/or community experience via REDCap (Harris et al., [48], Harris, [49]. The main questionnaire includes detailed questions on topics related to individual socio-demographic characteristics (e.g., age, gender identity (e.g. cisgender vs. transgender woman identity, inclusive of transfeminine identities^1^); sexual identities (e.g. Two-Spirit, Lesbian, Gay, Bisexual, Queer, and other terminologies to capture diverse sexual identities: 2SLGBQ + vs. heterosexual), drug use patterns, and structural factors including violence, work environment, criminalization and policing, sex work community mobilization, and access to health, substance use, social, and other services. In keeping with Canadian equity-oriented research standards, we reported gender identity and sexual identity as separate constructs, recognizing that gender and sexuality are distinct though sometimes overlapping domains of identity [50]. This approach aligns with federal usage of the inclusive acronym 2SLGBTQI+, where “2S” (Two-Spirit) is placed at the front to honour Indigenous traditions as the first individuals to experience sexual and gender diverse identities [51].

The questionnaire is updated regularly to capture emerging and changing priorities and needs within the community. Additionally, study visits include voluntary sexual health assessment and HIV/STI/HCV serology testing by a sexual health nurse at the study office or at a safe location identified by participants, along with referral/treatment as needed. Participants currently receive an honorarium of $80 at baseline and $65 CAD at each follow-up visit for their time and expertise and are offered connections to relevant health and social services at each visit as needed, alongside harm reduction supplies and other necessities.

### Study variables

#### Outcome

The primary outcome was defined as a binary (yes vs. no) variable based on the question, “do you currently own a take-home Narcan/naloxone rescue kit?” This question was added to the semi-annual questionnaire in March 2017 to reflect the changes in THN kit access and distribution in BC, which allowed for any person to access a kit without a prescription from a physician. This question was informed by community feedback regarding the importance of documenting sex workers’ ability to access and carry THN to respond to an overdose emergency.

#### Independent variables

Explanatory variables of interest were identified a priori based on previous literature and our conceptual framework. *Structural determinants in the sex work and drug use environment* included variables capturing housing precarity, homelessness, primary place of solicitation (indoor, independently vs. street/public space), primary place of providing services for sex work, and drug use in the context of sex work will be measured using a variable asking about using drugs with a client during a sex work transaction (yes vs. no). Additionally, *sex work community engagement* variables were considered such as utilization of sex worker-specific services defined as organizations which provide services to sex workers, such as health services and drop-in spaces, many of which are sex worker-led or have implemented sex worker-staff models. *Criminal legal system interactions* variables included negative police encounters while doing sex work and whether a participant experienced police-related barriers to harm reduction (e.g., difficulty accessing drugs, clean rigs, other drug equipment, rushing drug use, confiscation of drug equipment, money or drugs, ‘jacked up by cops’). *Access to health and substance use services* included variables about barriers to healthcare, enrollment in opioid agonist therapy (e.g., methadone, injectable buprenorphine, buprenorphine-naloxone, injectable hydromorphone, injectable diacetylmorphine, other prescription opioids for off-label addiction treatment) and accessed alcohol/drug treatment (yes vs. no).

Additionally, *individual characteristics* were included to characterize the study population and included the time-fixed demographics of age and racialization, which was coded as Indigenous identity (includes First Nations, Metis, and Inuit), Woman of Colour (e.g., Black, Asian, Middle Eastern), and White^2^. All other variables were assessed as time-updated measures with occurrences within the last 6 months prior to each study visit including identifying as a minoritized gender identity (e.g., transgender vs. cisgender) and identifying as a minoritized sexual identity (2SLGBQ + vs. heterosexual). *Drug use patterns* included types of drugs used (e.g., stimulants, opioids), route of administration (e.g., injection, non-injection), frequency of use (e.g., daily, less than daily), needing help with injecting (yes vs. no vs. N/A-no injection drug use), non-fatal overdose in the last 6 months (yes vs. no).

### Statistical analyses

Analyses were restricted to participants who reported having ever used criminalized injection or non-injection drugs (excluding cannabis and alcohol) and who completed an interview between March 2017 and March 2024. To address Aim 1, time series analysis was used to calculate semi-annual prevalence of THN ownership and plot the semi-annual proportion of participants who reported currently owning a THN kit from March 2017 to March 2024. To address Aim 2, structural and individual independent variables of interest were stratified by the outcome of THN ownership. To assess differences in THN ownership at participants’ first available observation, we used Pearson’s chi-squared test for categorical variables (or for small cell counts, Fisher’s exact test) and the Wilcoxon rank-sum test for continuous variables. We conducted logistic bivariate and multivariable generalized linear mixed-effects models (GLMM). To account for differences between individuals and the correlation within repeated measurements, we included random intercepts in the models, capturing individual-level variability. Listwise deletion was used to handle missing data. Structural variables that were hypothesized to be associated with the outcome and which had statistically meaningful relationships with the outcome in bivariate analysis were considered for inclusion in the full multivariable model, with collinearity assessed before final selection. We converted the subject-specific estimates to reflect population-level odds ratios using the marginal_coefs function from the GLMMadaptive R package [52]. Unadjusted odds ratios (OR) and adjusted odds ratios (AOR) with 95% confidence intervals (CI) were reported. All analyses were performed in R version 4.4.1.

## Results

**Fig. 1:**
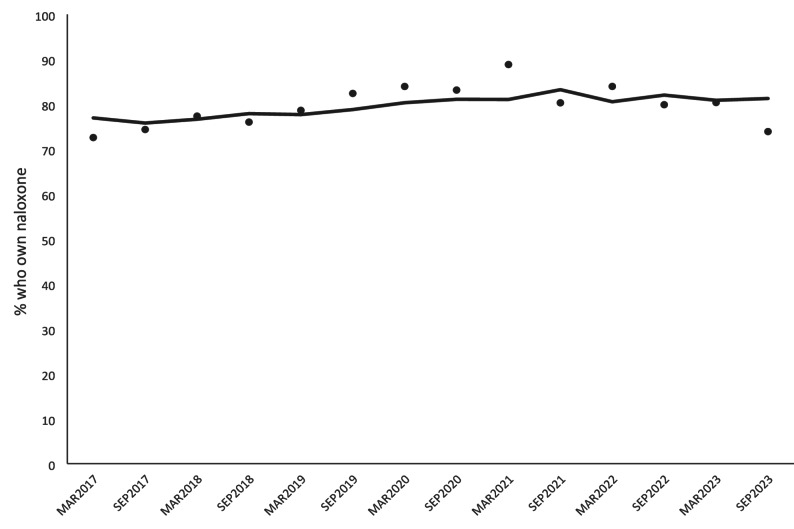
Semi-annual period prevalence of take-home naloxone (THN) ownership among sex workers (*N* = 506) AESHA, 2017–2024

Analyses included 506 sex workers who use drugs, who contributed 3007 interviews between 2017 and 2024 over a median number of 5 visits per participant. Almost three-quarters (73.7%, *N* = 373) reported currently owning a THN kit at the time of their first observation. 90.3% (*N* = 457) reported owning a THN kit over the entire study period. Over the 7-year study period, we observed high THN ownership, which ranged from 72.6% to 88.8% per semi-annual study period (Fig. 1).

At participants’ first observation (Table 1), the median age was 41 years old (interquartile range [IQR] = 33–48), and 54.2% (*N* = 274) reported identifying as a sexual minority. More than half (55.5%; *N* = 281) identified as Indigenous. With regards to structural determinants in the sex work and drug use environment, most (87.2%) reported precarious housing in the past six months, with 23.7% experiencing homelessness. Among those who owned THN, housing precarity was slightly higher (88.7% vs. 82.7%), but homelessness was lower (22.5% vs. 27.1%) compared to those who did not own THN. Sex workers who owned THN were more likely to have solicited in public (34.9% vs. 26.3%) and independent settings (30.3% vs. 24.8%) and more likely to have used drugs with clients during a sex work transaction (38.9% vs. 25.6%). They also reported higher use of sex worker-specific programs (58.2% vs. 43.6%) and were more likely to be enrolled in opioid agonist therapy (49.3% vs. 7.8%) and have accessed drug/alcohol treatment (42.4% vs. 28.6%). Those reporting owning THN also had higher rates of non-injection and injection drug use, particularly non-injection stimulants (65.2% vs. 52.6%), injection opioids (55.2% vs. 23.3%), and stimulants (41.0% vs. 21.1%). Daily opioid (49.6% vs. 18.8%) and stimulant use (42.6% vs. 27.1%) were also more common among THN owners.

**Table 1:** Study variables stratified by THN ownership among sex workers at baseline observation (*N* = 506) AESHA, 2017–2024

Characteristic	Total (%) (*N* = 506)	Total Missing (%)	Current THN Ownership	
			Yes (%) (*N* = 373)	No (%) (*N* = 133)
Temporal variables				
Year (median, IQR) (continuous)	2017 (2017–2018)		2017 (2017–2019)	2017 (2017–2018)
Precariously housed*	441 (87.2)	1 (0.2)	331 (88.7)	110 (82.7)
Homeless*	120 (23.7)		84 (22.5)	36 (27.1)
Primary place of solicitation*		12 (2.4)		
Outdoor/public space	165 (32.6)		130 (34.9)	35 (26.3)
Indoor (informal or formal)	34 (6.7)		23 (6.2)	11 (8.3)
Independent	146 (28.9)		113 (30.3)	33 (24.8)
N/A-didn’t solicit in last 6 months	149 (29.5)		98 (26.3)	51 (38.4)
Primary place of sex work service*		10 (2.0)		
Outdoor/public space	150 (29.6)		115 (30.8)	35 (26.3)
Formal/informal indoor	197 (38.9)		151 (40.5)	46 (34.6)
N/A-didn’t solicit in last 6 months	149 (29.5)		98 (26.3)	51 (38.4)
Used any drugs with client during a sex work transaction*^$^	179 (35.4)	51 (10.1)	145 (38.9)	34 (25.6)
Used sex worker-specific program*	275 (54.4)	1 (0.2)	217 (58.2)	58 (43.6)
Worked with other sex workers for safety*		4 (0.8)		
Yes	53 (10.5)		35 (9.4)	18 (13.5)
No	300 (59.3)		236 (63.3)	64 (48.1)
N/A-didn’t actively work in the last 6 months	149 (29.5)		98 (26.3)	51 (38.4)
Experienced violence from any negative police encounters*	75 (14.8)	6 (1.2)	57 (15.3)	18 (13.5)
Experienced any police-related barriers to harm reduction *	81 (16.0)	16 (3.2)	66 (17.7)	15 (11.3)
Experienced any healthcare barriers*	303 (59.9)	6 (1.2)	230 (61.7)	73 (54.9)
Enrollment in opioid agonist therapy*		13 (2.6)		
Yes	220 (43.5)		184 (49.3)	36 (27.1)
No	210 (41.5)		149 (40.0)	61 (45.9)
N/A-never used opiates	63 (12.5)		29 (7.8)	34 (25.6)
Accessed drug/alcohol treatment*	196 (38.7)	6 (1.2)	158 (42.4)	38 (28.6)
Overdosed*	70 (13.8)	5 (1.0)	55 (14.8)	15 (11.3)
Lifetime overdose experiences	291 (57.5)	9 (1.8)	233 (62.5)	58 (43.6)
Non–injection drug use†*	340 (67.2)	5 (1.0)	266 (71.3)	74 (55.6)
Injection drug use*	268 (53.0)	2 (0.4)	230 (61.7)	38 (28.6)
Individual characteristics				
Age, years (median, IQR)	41 (33–48)		41 (33–48)	40 (33–48)
Minoritized sexual identity*	274 (54.2)		208 (55.8)	66 (49.6)
Minoritized gender identity*	77 (15.2)		58 (15.6)	19 (14.3)
Racialization		1 (0.2)		
White	193 (38.1)		149 (40.0)	44 (33.1)
Indigenous	281 (55.5)		206 (55.2)	75 (56.4)
Woman of Colour^§^	31 (6.1)		17 (4.6)	14 (10.5)

* In the last 6 months

§ Due to low sample size these categories were combined for analysis and protection of confidentiality

$ Includes cannabis and alcohol

† Excluding cannabis and alcohol

In bivariate GLMM analysis (Table 2) odds of THN ownership were higher among those using drugs with sex work clients (Odds Ratio [OR] 1.43, 95% Confidence Interval [CI] 1.06–1.93), and those utilizing sex worker-specific programs (OR 1.37, 95%CI 1.06–1.77). Individuals currently enrolled in opioid agonist therapy (OR 2.09, 95%CI 1.54–2.84) and who accessed drug/alcohol treatment (OR 1.78, 95% CI 1.36–2.33) also had higher odds of THN ownership, whereas participants experiencing homelessness had lower odds of THN ownership (OR 0.39, 95% CI 0.27–0.58).

**Table 2:** Bivariate and multivariate correlates of THN ownership among sex workers, GLMM analysis (*N* = 506), AESHA, 2017–2024

Characteristic	Unadjusted odds ratio (95%CI)	Adjusted odds ratio (95%CI)
Structural determinants		
Precariously housed*	1.09 (0.87–1.37)	
Homeless*	0.63 (0.49–0.80)	**0.52 (0.37–0.73)**
Primary place of solicitation*		
Indoor (formal or informal)	0.82 (0.57–1.14)	
Independent	1.29 (0.99–1.67)	
N/A–didn’t solicit in the last 6 months	0.94 (0.74–1.19)	
Outdoor/public space (ref)		
Primary place of sex work service*		
Outdoor/public space	1.18 (0.93–1.50)	
Formal/informal indoor	1.22 (0.94–1.57)	
N/A–didn’t solicit in the last 6 months (ref)		
Used any drugs with client during a sex work transaction*	1.27 (1.04–1.56)	**1.27 (1.02–1.59)**
Used sex worker–specific program*	1.25 (1.04–1.49)	1.19 (0.94–1.50)
Worked with other sex workers for safety*		
Yes	0.61 (0.47–0.81)	
N/A–didn’t actively work in the last 6 months	0.81 (0.65-1.00)	
No (ref)		
Experienced any violence from community members/businesses*	1.16 (0.86–1.55)	
Experienced any physical/sexual violence from a client*	0.82 (0.62–1.09)	
Experienced violence from any negative police encounters*	1.00 (0.71–1.40)	
Experienced any police–related barriers to harm reduction*	0.97 (0.77–1.22)	
Experienced any healthcare barriers*	0.92 (0.78–1.08)	
Enrollment in opioid agonist therapy*		
Yes	1.72 (1.36–2.17)	**1.60 (1.29–1.98)**
N/A–never used opioids	0.48 (0.29–0.80)	0.62 (0.35–1.10)
No (ref)		
Accessed drug/alcohol treatment*	1.50 (1.24–1.82)	
Individual characteristics		
Age (per year older)	1.02 (1.00-1.03)	
Minoritized sexual identity*	1.20 (0.91–1.59)	1.17 (0.84–1.64)
Minoritized gender identity*	1.05 (0.75–1.48)	1.12 (0.73–1.71)
* Racialization*		
Indigenous ancestry	0.84 (0.61–1.15)	
Black or Woman of Colour^§^	0.37 (0.18–0.76)	
White (ref)		
* Drug use patterns*		
Overdosed*	1.36 (1.05–1.76)	
Lifetime overdose experiences	1.27 (0.99–1.64)	0.99 (0.74–1.32)
Non–injection drug use†*	1.47 (1.23–1.75)	
Injection drug use*	1.80 (1.48–2.20)	**1.70 (1.35–2.14)**
Temporal characteristics		
Year (continuous)	1.06 (1.01–1.12)	

* In the last 6 months

§ Due to low sample size these categories were combined for analysis and protection of confidentiality

† Excluding cannabis and alcohol

Bolded results are statistically significant

In the multivariable GLMM analysis (Table 2), 480 participants contributed 2519 observations (83.8% of the study sample). After adjustment, injection drug use (Adjusted Odds Ratio [AOR] 1.70, 95% CI 1.35–2.14), enrollment in opioid agonist therapy (AOR 1.60, 95% CI 1.29–1.98), and having used drugs with a client during a sex work transaction (AOR 1.27, 95% CI 1.02–1.59) were associated with higher odds of THN ownership; whereas homelessness was associated with lower odds of THN ownership (AOR 0.52, 95% CI 0.37–0.73). Having used sex work-specific programs was marginally associated with higher odds of THN ownership (AOR 1.19, 95% CI 0.94–1.50, *p* = 0.144).

## Discussion

In this prospective, community-based cohort study, 90% of sex workers who use drugs reported owning THN at least once over a 7-year follow-up period (2017–2024), and the proportion who owned THN during each 6-month study follow-up period ranged from 72.6 to 88.8%, suggesting very high access to THN among this population. Participants enrolled in opioid agonist therapy, who used drugs with clients during sex work transactions, and accessed sex worker-specific services and were more likely to possess THN, whereas those facing homelessness had lower odds; however, after inclusion of individual-level factors, accessing sex worker-specific services was marginally associated. Our findings indicate that THN ownership reflects a structural marker of inclusion or exclusion within harm reduction and substance use treatment systems—influenced by interlocking systems of criminalization, precarious housing, and gendered violence that influence vulnerability to overdose as well as access to lifesaving tools and services. These findings suggest that differential inclusion in care and intervention programmes may undermine the realization of the right to health as articulated in the NSWP Consensus Statement on Sex Work, Human Rights and the Law, particularly its emphasis on equitable, non-discriminatory access to health services and protection from preventable harms including overdose (Global Network of Sex Work Projects, [53]). This study is one of few to measure and assess patterns and structural correlates of naloxone ownership among sex workers who use drugs, which is an important and under-researched step in the naloxone cascade [15] and provides novel insights contributing to the global literature on the overdose prevention strategies and tools implemented by sex workers. By leveraging seven years of longitudinal data, this study uniquely captures how evolving structural conditions—such as shifts in policy, pandemic-era disruptions, and escalating overdoses—shape patterns of equitable access to a life-saving overdose prevention tool among sex workers who use drugs.

We documented much higher levels of naloxone ownership compared to prior research on the naloxone cascade with people who use drugs in other major North American cities [15, 24, 54–56], and a systematic review which included studies from North America, Norway, and Scotland that reported pooled prevalence of THN ownership of 57% [57]. Our study results more closely reflect THN ownership prevalence among adults who use drugs in BC (70.7%) [58], where significant public health efforts have invested in overdose response efforts including naloxone distribution and training. In our study, odds of owning THN were high among those enrolled in opioid agonist therapy. Opioid agonist therapy is associated with reduced mortality among those who use opioids [59]. However issues with treatment retention, opioid tolerance, and insufficient dosing can lead to continued use of opioids sourced from the unregulated drug supply [60, 61]. Given the unpredictability and toxicity of drugs in the unregulated supply, THN is a valuable tool to address overdose. While THN was not previously part of clinical guidelines for the treatment of opioid use disorder, discussions and referral to THN programs are now recommended to be routinely offered as part of standard care in BC (British Columbia Centre on Substance Use et al., [62]; *Changes to B.C.’S Opioid Use Disorder Guidelines|BC Pharmacy Association*, [63]; Kleinman, [64].

In this study, sex workers who used drugs with a client during a sex work transaction had higher odds of owning THN. Sex workers who use drugs face distinctive hurdles as an equity-denied group facing overlapping criminalization and marginalization, as there are no settings in which both sex work and drug use are fully decriminalized [11]. Regulatory and legal frameworks that criminalize sex work and drug use preclude sex workers from accessing THN as part of workplace occupational protections [65, 66]. Our results bolster previous qualitative research involving sex workers who use drugs in their management of the overdose risk environment for sex work clients [67]. Sex workers in the study described how they provided supervised consumption for clients, informally offering peer witnessing to clients among other safer drug practices (e.g., safer injecting education). THN ownership underscores a proactive approach to safety that is in alignment with other harm reduction principles (e.g., carrying condoms) that prioritizes sex workers safety for themselves and others, even in environments where stigma and legal restrictions exist. These strategies also reflect sex workers’ assertion of their fundamental right to health and occupational safety, demonstrating how, despite systemic exclusion, they actively create protective practices that advance both individual and community well-being. Such efforts are consistent with the human rights framework articulated in the NSWP Consensus Statement, which positions access to health care and occupational safety as central to the realization of health equity (Global Network of Sex Work Projects, [53]).

Participants who accessed sex worker-specific programs had marginally higher odds of owning THN kits compared to those who did not access this tool. Sex workers experience many barriers to receiving healthcare and supports due to policing practices, stigma, and incompatibility of service offerings with sex workers’ needs [68–70]. Sex worker-specific programs such as peer-based education, outreach, and safe, dedicated drop-in services can facilitate the delivery of a range of health and support services in culturally safe and accessible manner (i.e., extended hours, mobile outreach to work environments). While these programs are sparse and vastly unfunded, especially in settings where sex work is criminalized, current evidence suggests that these models can successfully deliver essential programming and interventions to support health and safety needs [35, 71–73], and should be expanded to include and/or scale-up THN training and distribution in contexts where sex workers face high overdose risk. Scaling up such models to include and expand THN training and distribution is essential to advance equity and uphold the right to health among sex workers.

Additionally, our study findings indicate that sex workers facing homelessness faced significantly lower odds of owning THN. Populations facing homelessness and other forms of housing precarity are known to face enhanced barriers to services and may be forced to use drugs in public settings, both of which may increase overdose risk [26, 74, 75]. Access to safe and affordable housing is crucial for addressing health disparities [31, 76], yet sex workers face barriers in obtaining and maintaining suitable housing due to the criminalization and stigma associated with sex work [77, 78] and among sex workers who use drugs, drug use as well. In BC, as in other settings, “street sweeps” have been intensifying, displacing alarming numbers of community members from public settings and confiscating and discarding their personal belongings as refuse, including essential survival and health items such as THN [79, 80]. For sex workers who use drugs, street sweeps entrench poverty and deepen exclusion from essential harm reduction services. Scaling-up access to safe, affordable, dignified and non-discriminatory housing, as well as cessation of “street sweeps” is therefore not only a public health necessity but also a human rights imperative. Such interventions align with intersectional harm reduction approaches that address structural inequities and prioritize the safety of equity-denied communities.

### Strengths and limitations

Our study describes structural determinants and time trends in ownership of THN amidst an environment of elevated morbidity and mortality rates linked to the unregulated, criminalized drug supply. Notably, our findings address a critical gap highlighted in a recent global systematic review, which underscored the paucity of academic literature capturing the multifaceted experiences of sex workers who use drugs, particularly in contexts where drug use remains criminalized [11]. This study also underscores the critical role of community-based, sex worker-focused programs and services as strategic platforms for training and dissemination of community-based overdose prevention tools. Our study also has limitations. As with all questionnaire-based research, potential biases in measurement tools could have resulted in under-reporting of stigmatized and criminalized behaviors. Additionally, our reliance on self-reported data, particularly for more rare exposures, may be subject to recall bias, which would have biased findings towards the null [81–84].

## Conclusion

In this study, most sex workers who use drugs owned THN, which was associated with enrollment in opioid agonist therapy, drug use with clients, and utilization of sex worker-specific programming. Results were slightly attenuated for enrollment in opioid agonist therapy in the fully adjusted model which included individual-level factors such as recent injection drug use. Our study found an association between drug use with a client during a sex work transaction and THN ownership which suggests that sex workers may be proactively taking measures to mediate overdose-related risks for themselves and others in the community in the context of their work. To advance sex workers’ right to health, we recommend sustained investment in sex worker-specific support services that support linkages to treatment and include THN distribution and training, alongside upstream structural occupational work safety and overdose prevention measures.

## Data Availability

Data for this study are not publicly available for legal and ethical reasons, as this study involves sensitive data collected with a highly criminalized and stigmatized population of marginalized women. Under our current ethical approvals by the Providence Health Care—University of British Columbia (PHC-UBC) Institutional Research Ethics Board, de-identified data can be made available upon reasonable request and pending ethical approval. Please submit all requests to initiate the data access process to the corresponding author and PHC-UBC REB at [ubc.all-reb@ubc.ca](mailto: ubc.all-reb@ubc.ca) .

## Footnotes

1 The authors recognize the fluidity of gender and while cis and transfeminine gender identification was part of baseline enrollment criteria people can identify in different ways at different times.

2 Given the low population of Black people in the Province of BC (less than 2%), the categories Black and Women of Colour were aggregated for analysis due to low sample size and the need to protect participant confidentiality.
